# Building the Precision Medicine for Mental Disorders via Radiomics/Machine Learning and Neuroimaging

**DOI:** 10.3389/fnins.2021.685005

**Published:** 2021-06-15

**Authors:** Long-Biao Cui, Xian Xu, Feng Cao

**Affiliations:** ^1^Department of Radiology, The Second Medical Center, Chinese PLA General Hospital, Beijing, China; ^2^Department of Clinical Psychology, School of Medical Psychology, Fourth Military Medical University, Xi'an, China; ^3^The Second Medical Center, National Research Center for Geriatric Disease, Chinese PLA General Hospital, Beijing, China

**Keywords:** radiomics, machine learning, mental disorders, magnetic resonance imaging, schizophrenia

## Introduction

A pressing need of diagnostic, predictive, and prognostic markers exists in clinical settings. Unfortunately, in spite of evidence for underpinnings in mental disorders over the past decade, biologically based markers in reflection to the physiology for guiding clinical practice have obviously lagged behind. On the one hand, the updated Diagnostic and Statistical Manual of Mental Disorders remains to emphasize how mental disorders are expressed, although it provides a model that helps clinicians to perform better diagnosis and follow-up care (Kupfer et al., 2013), like cholesterol and blood pressure measurement. On the other hand, as the 23rd edition of the “Clinical Handbook of Psychotropic Drugs” has said, “we can provide current evidence-based and clinically relevant information to optimize patient care,” but the information is derived from randomized controlled trials and leading clinical experts, etc. Therefore, clinical practice requests guidance of some objective, quantitative, and specific biomarker, reflecting its neurobiological substrates for diagnosis and treatment selection.

To this end, machine learning methods, as demonstrated by a sizable number of recent neuroimaging studies, hold great promise for improving the diagnosis, treatment, and prediction of prognosis in psychiatric domains, which will have an effect on personalized medicine. The term “machine learning” was coined in 1959 by Arthur Samuel (Samuel, 1959), and it is a science of the artificial intelligence, showing an evident capacity to reveal relationships between different variables used for classification (Tandon and Tandon, 2018). Furthermore, radiomics is a newly developed method to obtain high-dimensional features that might be options used for machine learning analysis. This Research Topic “Machine Learning in Neuroscience, Volume II” in “Frontiers in Neuroscience” provides new study strategy and applies radiomics/machine learning and distinct neuroimaging in mental disorders. Transforming existing clinical pathways toward optimizing care for the specific needs of each psychiatric patient, the significance is to achieve better diagnosis, treatment, and prognosis of mental disorders using radiomics/machine learning.

The field of psychiatry research remains a focus of medicine; in particular, mental health has arrived on the global health agenda. Currently, PubMed comprises more than 10 citations for literature involving radiomics and mental disorders, and efforts to develop radiomics/machine learning-based objective means have intensified for autism spectrum disorder (Chaddad et al., 2017), attention-deficit/hyperactivity disorder (Sun et al., 2018), schizophrenia spectrum and other psychotic disorders (Cui et al., 2018, 2021; Gong et al., 2020; Park et al., 2020; Xi et al., 2020), bipolar and related disorders (Wang et al., 2020), mild cognitive impairment, and Alzheimer's disease (Kai et al., 2018; Li et al., 2018; Ranjbar et al., 2019; Huang et al., 2020). Radiomics/machine learning enables the neuroimaging data of mental disorders to be extracted for improving clinical decision support (Wang et al., 2019).

Due to the excellent performance of radiomics analysis for feature selection and classification, it is regarded as the bridge between medical imaging and personalized medicine (Lambin et al., 2017). However, a critical issue is related to radiomics analysis in non-cancer field. In spite of a lack of lesions for conventional feature extraction, neuroimaging-based measures are features that could be extracted in radiomics/machine learning study. Taking schizophrenia as an example, previous studies on this topic illustrate the potential value in the application of radiomics/machine learning methods to disease definition and diagnosis and prediction of response to antipsychotics (APs) or electroconvulsive therapy (ECT).

## Radiomics and Magnetic Resonance Imaging in Schizophrenia

Diagnosis and treatment of schizophrenia are pivotal clinical issues that need to be solved urgently. In a recent review, Kraguljac et al. (2021) highlighted and discussed the neuroimaging biomarkers in schizophrenia. Magnetic resonance imaging (MRI), as a non-invasive neuroimaging method, has been widely used in the study of schizophrenia. As we commented on a meta-analysis of the association of clinical and demographic characteristics and magnetic resonance spectroscopy in schizophrenia (“Targeting the Whole Clinical Course of Schizophrenia With Magnetic Resonance Imaging,” https://jamanetwork.com/journals/jamapsychiatry/fullarticle/2778479), MRI combined with radiomics/machine learning could be the most important approach in schizophrenia research, involving predicting transition from clinical high risk to psychosis, providing evidence of macroscale neural mechanisms, delving into the nature behind symptoms, facilitating diagnosis and subtyping, predicting treatment response, detecting psychopharmacological effects, and guiding neuronavigation of neuromodulation, thereby managing very-late-onset schizophrenia-like psychosis. MRI-based studies are promising for clinical translation (Jiang et al., 2020).

It is of no doubt that we took the precision medicine view more seriously with the advent of radiomics/machine learning in the field of schizophrenia research (Wang et al., 2019). The number of publications on radiomics/machine learning via MRI has increased to five until this year, including one regression analysis (Gong et al., 2020) and four classification analyses (Cui et al., 2018, 2021; Park et al., 2020; Xi et al., 2020) (Table 1). There are two well-done MRI studies from Xi et al. (2020) and Gong et al. (2020), respectively. They used radiomics features on structural MRI (sMRI) to predict response to APs plus ECT. In the first study, the group of Yi-Bin Xi and colleagues extracted radiomics features from the regions of interest (ROIs) with differences of gray matter volume between responders and non-responders (Xi et al., 2020). Specifically, voxel-wise gray matter volume was compared between responders and non-responders, and then, 11 ROIs identified in the previous step entered first-order statistics feature extraction and classification analysis. A leave-one-out cross-validation (LOOCV) framework and support vector machine (SVM) was used to perform pattern classification analysis. This study built a fusion logistic regression model (LRM) with the least absolute shrinkage and selection operator (LASSO) with an accuracy of 93.18%, and the fixed features were from the right anterior cingulum, left supramarginal gyrus, and right hippocampus. In the second study, the group of Gong et al. (2020) examined whether the combination of gray and white matters can predict the outcome using sMRI and diffusion tensor imaging. They selected first-order statistics radiomics features from regions (gray and white matters) with strong electric field distribution under ECT in this regression analysis study. The prediction process was performed with a support vector regression model based on a LOOCV framework. Features in the left inferior frontal gyrus, right superior temporal gyrus, left temporal pole, right insula, and fibers connecting the frontal and temporal lobes were used in the final support vector regression model. The majority of ECT studies thus far has focused on identification of treatment response biomarkers in major depressive disorder. These are interesting studies that seek to predict treatment response in patients with schizophrenia undergoing electroconvulsive therapy. Based on the similar radiomics features, Park et al. (2020) used bilateral hippocampal subfields to differentiate patients with schizophrenia from healthy controls. ROIs were automatically segmented, and various combinations of classifiers (LRM, extra-trees, AdaBoost, XGBoost, or SVM) were trained, yielding an accuracy of 82.1%. These findings on the basis of structural differences with biological significance thus offer the potential to add new information to the literature.

**Table 1:** Radiomics/machine learning and MRI in schizophrenia.

**Articles**	**Subject number**	**MRI**	**Features selected**	**Accuracy**	**Sensitivity**	**Specificity**
**Identifying patients**						
Park et al. (2020)	Training: Pat = 60/Con = 46 Testing: Pat = 26/Con = 20	sMRI	30 radiomics features from the bilateral hippocampal subfields	82.1%	76.9%	70%
Cui et al. (2018)	Training: Pat = 52/Con = 66 Testing: Pat = 56/Con = 55	fMRI	32 connections of the whole brain	87.09%	86.79%	87.22%
**Predicting treatment response**						
Cui et al. (2021)	Training: R = 47/*N* = 38 Testing: R = 41/*N* = 22	sMRI/fMRI	Nine functional connections and three cortical features	85.03%	92.04%	80.23%
Xi et al. (2020)	Training: R = 22/*N* = 22 Testing: R = 6/*N* = 7	sMRI	Three gray matter radiomics features	93.18%	95.45%	90.91%

*Search terms: “schizophrenia and radiomics”*

*Con, controls; N, non-responder; Pat, patient; R, responder*.

In addition to conventional features, another two studies considered abnormal functional connectivity as features (Cui et al., 2018, 2021). In a disease identification study, a total of 137 connections determined by functional MRI (fMRI) were detected between patients with schizophrenia and healthy controls (Cui et al., 2018). Then, they reduced to 32 using the LASSO binary LRM. The accuracy of detecting patients was 87.09%. One of the strengths of this study is the two types of cross-validation (CV), i.e., intra- and inter-data set CV. In an early treatment response prediction study, both functional connectivity and cortical measures with group difference were used to obtain baseline features (Cui et al., 2021). They used CV-LASSO to conduct feature selection and dimension reduction and constructed an SVM model to predict response to treatment. The combined features obtained an outstanding accuracy with 85.03%. Likewise, biologically meaningful group differences of MRI reflect the pathophysiology of schizophrenia in relation to diagnosis and treatment. Although these two studies included non-conventional radiomics features, MRI analyses produce tens of thousands of functional connections and cortical measurements. In line with radiomics, high-throughput mining of quantitative features from medical imaging, we can call it the radiomics strategy in schizophrenia research.

Nevertheless, dozens of MRI studies using machine learning are emerging in schizophrenia. Many of them are characterized by large international multicenter samples (Chen et al., 2020), multimodal MRI fusions (Lei et al., 2020), elegant machine learning models (Rozycki et al., 2018), considerable accuracy with high generalizability (Koutsouleris et al., 2018), or enhanced understanding of brain circuits that can serve as potential biomarkers (Zhao et al., 2020). For this reason, MRI-based machine learning approaches may offer better individual-level diagnostic and predictive value in mental disorders (Keshavan et al., 2020).

## Discussion

MRI-based radiomics/machine learning studies hold several strengths with regard to schizophrenia, e.g., having biological underpinnings (structure/function), extension of treatment prediction (APs/ECT), and validation methods (intra-/inter-data set CV). However, many critical challenges exist in this field from both clinical and research perspectives. First, most current classification studies treat a clinical diagnosis as the gold standard; however, with MRI or psychopathology, some recent unsupervised (Jauhar et al., 2018; Matsubara et al., 2019) or supervised (Jacobs et al., 2021) machine learning studies have tended to explore transdiagnostic characteristics of mental disorders and try to break the boundary of classic diagnosis and establish bioinformation-based disorder classification. Second, many novel machine learning models, such as generative adversarial networks (GANs), have been applied to large multicenter MRI data in this field (Zhong et al., 2020; Ren et al., 2021). GANs contribute a lot to improving reproducibility of radiomics features across manufacturers and increasing diagnostic accuracy (Marcadent et al., 2020). The use of “radiomics” combining novel machine learning models is considered an initiative and an important development over prior work in the precision medicine of mental disorders.

Driven by the need for better management of patients, as well as advances in neuroimaging-based machine learning approach, a quest for accurate detection of convention to illness, identification of patients, and prediction of treatment response and outcome is noted. MRI-based radiomics/machine learning researchers should promote the generalizability of findings across patients and pave the way to facilitate the guidance of clinical decision making by means of these findings.

## Author Contributions

L-BC conceptualized and wrote the first draft of the manuscript. All authors contributed to the article and approved the submitted version.

## Conflict of Interest

The authors declare that the research was conducted in the absence of any commercial or financial relationships that could be construed as a potential conflict of interest.
